# Stem Cell Therapy in Critical Limb Ischemia

**DOI:** 10.7759/cureus.41772

**Published:** 2023-07-12

**Authors:** Madhan Jeyaraman, Somumurthy Nagarajan, Nicola Maffulli, Packkyarathinam R.P, Naveen Jeyaraman, Arulkumar N, Manish Khanna, Sankalp Yadav, Ashim Gupta

**Affiliations:** 1 Orthopaedics, ACS Medical College and Hospital, Dr. MGR Educational and Research Institute, Chennai, IND; 2 Regenerative Medicine, Indian Stem Cell Study Group Association, Lucknow, IND; 3 Regenerative Medicine, Datta Meghe Institute of Higher Education and Research, Wardha, IND; 4 Biotechnology, School of Engineering and Technology, Sharda University, Greater Noida, IND; 5 Orthopaedics, South Texas Orthopaedic Research Institute, Laredo, USA; 6 Orthopaedic Rheumatology, Dr. Ram Manohar Lohiya National Law University, Lucknow, IND; 7 Orthopedics, School of Medicine and Surgery, University of Salerno, Fisciano, ITA; 8 Orthopaedics, San Giovanni di Dio e Ruggi D’Aragona Hospital, Hospital of Salerno, Salerno, ITA; 9 Orthopedics, Barts and the London School of Medicine and Dentistry, London, GBR; 10 Orthopedics, Keele University School of Medicine, Stoke-on-Trent, GBR; 11 Regenerative and Interventional Orthobiologics, Dr. Ram Manohar Lohiya National Law University, Lucknow, IND; 12 Orthopaedics, Autonomous State Medical College, Ayodhya, IND; 13 Internal Medicine, Shri Madan Lal Khurana Chest Clinic, New Delhi, IND; 14 Regenerative Medicine, Future Biologics, Lawrenceville, USA; 15 Regenerative Medicine, BioIntegrate, Lawrenceville, USA; 16 Regenerative Medicine, Regenerative Orthopaedics, Noida, IND

**Keywords:** ischemia, amputation rates, mononuclear cells, critical limb ischemia, stem cell

## Abstract

Critical limb ischemia (CLI), a serious outcome of peripheral artery disease, is frequently associated with morbid outcomes. The available treatment modalities do not provide satisfactory results, leading to marked morbidities such as joint contracture and amputations, resulting in a high economic burden. The peripheral vascular disease tends to cause more morbidity in patients with diabetes and atherosclerosis, given the pre-existing compromised perfusion of medium and small vessels in diabetic patients. With surgical procedures, the chance of vascular compromise further increases, inducing a significantly greater rate of amputation. Hence, the need for nonsurgical treatment modalities such as stem cell therapy (SCT), which promotes angiogenesis, is warranted. In CLI, SCT acts through neovascularization and the development of collateral arteries, which increases blood supply to the soft tissues of the ischemic limb, providing satisfactory outcomes. An electronic database search was performed in PubMed, SCOPUS, EMBASE, and ScienceDirect to identify published clinical trial data, research studies, and review articles on stem cell therapy in critical limb ischemia. The search resulted in a total of 2391 results. Duplicate articles screening resulted in 565 articles. In-depth screening of abstracts and research titles excluded 520 articles, yielding 45 articles suitable for full-text review. On review of full text, articles with overlapping and similar results were filtered, ending in 25 articles.

SCT promotes arteriogenesis, and bone marrow-derived mesenchymal stromal cells produce significant effects like reduced morbidity, improved amputation-free survival (AFS ) rate, and improved distal perfusion even in "no-option" CLI patients. SCT is a promising treatment modality for CLI patients, even in those in whom endovascular and revascularization procedures are impossible. SCT assures a prolonged AFS rate, improved distal perfusion, improved walking distances, reduced amputation rates, and increased survival ratio, and is well-tolerated.

## Introduction and background

Critical limb ischemia (CLI) is a serious and potentially fatal form of peripheral artery disease (PAD), with low evidence of therapeutic success with the available treatment modalities [1,2]. Thromboembolism and atherosclerosis may induce CLI with short-term mortality and adverse cardiovascular effects [3,4]. The most common manifestation of CLI is observed in smokers with severe leg pain, ulcers, or gangrenous toe. Despite advances in treatment, including surgical and interventional radiological intervention, patients still undergo major or minor amputation [5,6].

Approximately 20% of Americans over 65 and 50% of patients aged 75 are diagnosed with PAD [7], with eight million Americans diagnosed with limb ischemia [8]. In India, there is a double or triple incidence of PAD and/or CLI, of which 10-40% require amputation [9] and gangrene [10]. The use of stem cell therapy for this purpose is beneficial by increasing the number of new cells producing growth factors and stimulating neoangiogenesis [11-13].

Risk factors of PAD are a) Ankle-brachial index (ABI) <0.09, greater in non-Hispanic blacks, b) hypertension, c) dyslipidemia, d) raised CRP in asymptomatic individuals, e) hyperviscosity and hypercoagulation state, f) hyperhomocysteinemia, and g) chronic renal insufficiency [14-17]. Revascularization is the cornerstone of treatment whenever possible, yet amputations and death remain common. Significant major amputation rates in the range of 10-40% have been observed in these patients, particularly those with unsuccessful revascularization or "no-option" CLI (NO-CLI) [12]. Exploring newer techniques for the revascularization of these ischemic limbs is essential. Cell-based therapeutics have emerged as a new area in this field, with bone marrow-derived mesenchymal stem cells (BM-MSCs) currently viewed as a promising and potentially newer therapeutic approach. Numerous research, including randomized trials, nonrandomized trials, and uncontrolled studies, have demonstrated the efficacy of stem cell therapy in CLI patients [11-13]. However, given study variability, small sample numbers, and a lack of large-scale placebo-controlled research, acceptance of this modality of therapy as the standard of care remains debatable. Transplantation of autologous BM-MSCs has also been tested in terms of several implantation techniques, either intramuscular (IM) injection, intra-articular (IA) injection, or combination, and has yielded essentially identical results [12]. Generally, stem cell-based therapy is safe and effective, with modest and mostly temporary adverse responses associated with local implantation. Moreover, preconditioning methods and prolonged growth factor release using bioactive microspheres may improve the therapeutic efficacy of cell treatment.

The current review aims to evaluate and assess the clinical data and findings regarding the use of stem cell therapy in CLI patients with better therapeutic outcomes.

## Review

Methods

Search Criteria

**Figure 1:**
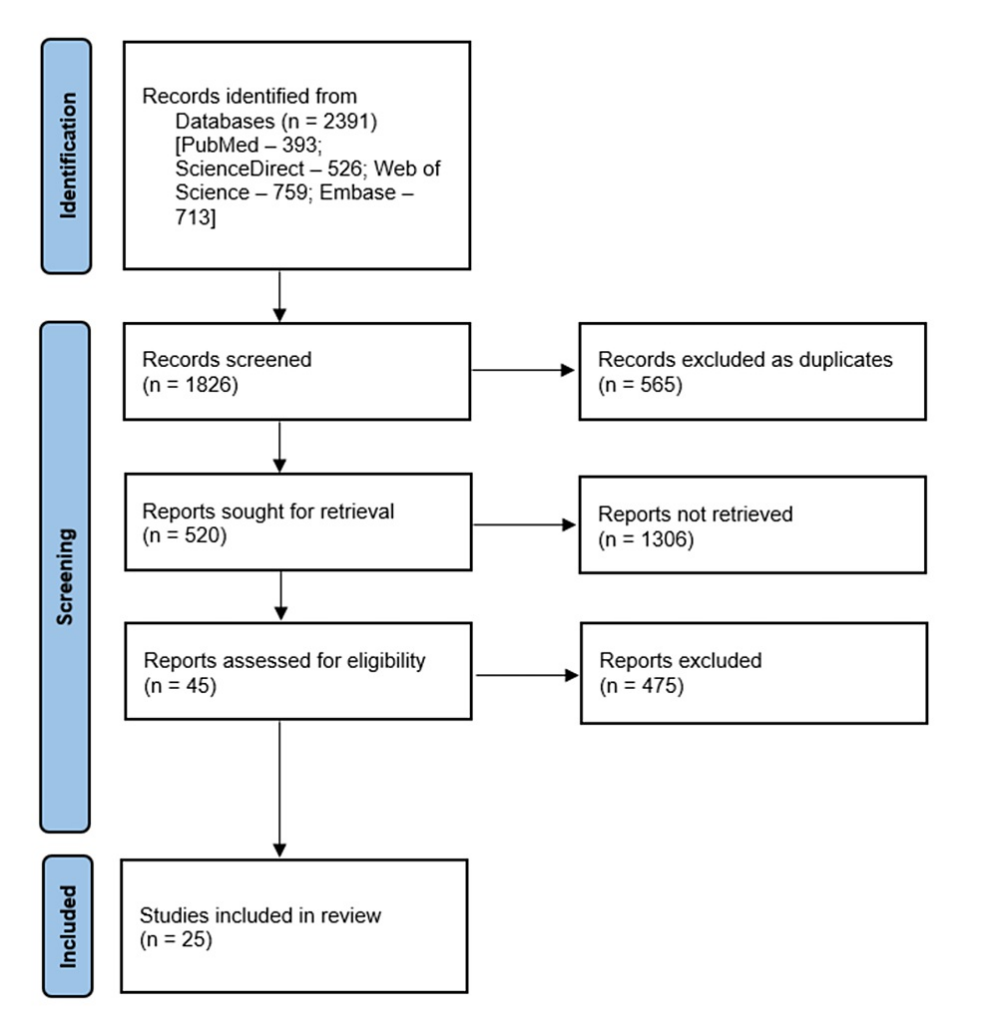
Literature search per PRISMA guidelines PRISMA-Preferred Reporting Items for Systematic Reviews and Meta-Analyses

A comprehensive search was conducted in PubMed, EMBASE, SCOPUS, and Web of Science, resulting in 1865 articles. The search was performed to identify articles having data on peripheral arterial obstructive disease (PAD), critical limb ischemia, angiogenesis, limb loss, and amputations together with BOOLEAN terms "OR", "AND," and "NOT". The search strategy was conducted with adherence to the Preferred Reporting Items for Systematic Reviews and Meta-Analyses (PRISMA) guidelines (Figure 1). An additional search was conducted using the reference articles of the primary results to include studies left out.

Inclusion Criteria

Only articles published in English were included and reviewed by the first author to identify relevant studies. We evaluated all potentially eligible studies through an in-depth review and consideration of the full text. Reference lists and relevant publications in the articles' bibliography were also searched. Data from clinical trials that mainly used stem cells in CLI patients were included.

Exclusion Criteria

Studies with primarily targeted indications other than CLI, such as carotid disease, aortic aneurysmal disease, inflammatory disease, cancer, nonvascular disease, intracranial vascular disease, and chemotherapy treatment, were excluded from the study.

Results

The initial database search yielded 2391 results, of which 393 studies were from PubMed, 526 from ScienceDirect, 759 from Web of Science, and 713 from EMBASE. Duplicate articles screening resulted in 565 articles. In-depth screening of abstracts and article titles excluded 520 articles, yielding 45 articles suitable for full-text review. On review of full text, articles with overlapping results were filtered, ending up in 25 articles included in this study. Some of the significant results from individual studies are as follows:-

Hybrid Revascularization

A CLI patient may undergo a combination of endovascular and open surgery with decreased extensive invasive procedures and shorter duration. Endovascular surgery consists of inflow, outflow, or a combination of both; it is performed percutaneously using a cross-over technique in the ipsilateral or contralateral femoral artery. Dougherty et al. studied 125 patients with hybrid therapy and found limited effectiveness in non-ambulatory patients in old age, and if multiple co-morbidity were present, those patients needed therapeutical angiogenesis [18].

Vascular Stem Cells Biology

Thompson et al. reported that embryonic stem cells (ESCs) retain the ability to perpetually regenerate themselves while still possessing the capacity to differentiate into any form of human body cell [15]. The inner cell mass (ICM), as the name suggests, forms the innermost cellular component of the embryonic cell blastocyst and gives birth to primitive endoderm and epiblast, which in turn are composed of the ectoderm, mesoderm, and endoderm main germ layers, throughout the physiological process of embryogenesis [19].

Vasculogenesis and angiogenesis are two different processes. Vasculogenesis is the process where new blood vessels form from endothelial progenitors and is primarily an embryonic process; the resultant capillaries are tiny and, as a result of the Hagen-Poiseuille rule, cannot appropriately replace larger ones blocked in CLI. Arteriogenesis, also known as collateral growth, is the process by which pre-existing arterioles present collaterally are transformed into functioning collateral arteries. Human mesenchymal stromal cells derived from bone marrow trigger cellular events and paracrine processes, which have been shown to assist arteriogenesis [19].

Elser et al. and Jaminon et al. demonstrated that ischemia increases plasma levels of activated cell cytokines such as thrombopoietin and soluble kit-ligand (sKitL) as well as progenitor cell cytokines like granulocyte-macrophage colony-stimulating factor (GM-CSF) and erythropoietin [20,21]. The release of stromal cell-derived factor-1 (SDF-1) by thrombopoietin and sKitL in ischemic limbs may enable revascularization by supporting hemangiocyte mobilization. In addition, immature vascular smooth muscle cells (VSMCs) perform a vital role in the proliferation and migration to the site, followed by creating extracellular matrix (ECM) and vascular wall components such as collagen, elastin, and proteoglycans. All these contribute to an efficient process of vascular morphogenesis [21].

Tateishi-Yuyama et al. performed a pilot study with 25 patients suffering from unilateral ischemic leg who received injections of bone marrow-mononuclear cells (BM-MNCs) in the gastrocnemius of the ischemic limb. Twenty-two patients having bilateral ischemic legs underwent injection of BM-MNCs in one leg and peripheral mononuclear cells in the other. Ankle-brachial index (ABI) was significantly improved in those who had received BM-MNCs at 24 weeks. Autologous implantation of BM-MNCs is effective for therapeutic angiogenesis due to increased progenitor cell consistency, angiogenic factors cytokines, and a cluster of differentiation 34 (CD-34) cells [22].

Stromal Cells for Angiogenesis, Vasculogenesis, and Wound Healing

**Figure 2:**
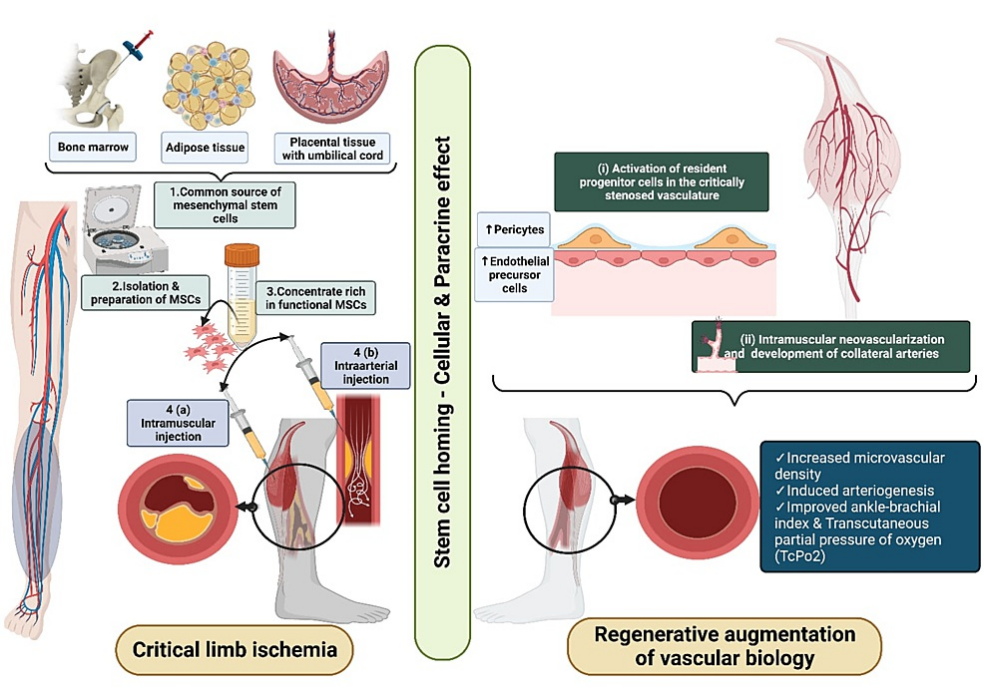
Regenerative principles of critical limb ischemia Picture courtesy Dr. Madhan Jeyaraman

Increasing blood flow can theoretically be attained by increasing angiogenesis either pharmacologically or biologically, allowing therapeutic angiogenesis. Based on in-vitro studies, nitrous oxide plays a major role in bone marrow mobilization and endothelial progenitor cell release. Hyperbaric oxygen therapy (HBOT) increases nitrous oxide in cerebral cortex tissue, pulmonary tissue, and neutrophils, in turn increasing circulating progenitor cells, CD34, and myeloid marker cluster of differentiation 14 (CD14) [23-26]. Hence, HBOT is safe to enhance the mobilization of bone marrow (BM) derived progenitor cells into the circulation with minimal side effects Figure 2.

Stromal Cell Mobilization

Hematopoietic stem cells (HSCs) are harbored in the BM, and several chemokines and cytokines promote HSC mobilization to the peripheral circulation. This mobilization of stem cells results from the complex interactions between many important cell proteases, ligands, and receptors in the extracellular milieu [19]. A dose-dependent association exists between the increased number of endothelial progenitor cells (EPCs) in the peripheral circulation and the injection of granulocyte colony-stimulating factor (G-CSF) and GM-CSF. In addition, the chemokine receptor (CXC) leads to the mobilization of EPCs, which increases matrix metallopeptidase 9 (MMP-9) signaling in BM [27,28].

GM-CSF mobilizes fewer cells than G-CSF, so it is rarely utilized in place of G-CSF. In general, vascular endothelial growth factor (VEGF), fibroblast growth factors, and factors generated from stromal cells can promote EPC recruitment. Statins, parathormones, and cell-mobilizing ligands can be used alone or in conjunction with G-CSF. Using BM-derived cells (BMDCs) in stem cell treatment has demonstrated reasonable safety. However, difficulty was seen in connection to the collection and mobilization of cells. A one-day culture of activated dendritic cells (DC) allowed us to observe the development of EPC-enriched stem cells [29].

Skeletal muscle satellite cells are another potential source of stem cells requiring further research and exploration [30]. After birth, muscles harbor precursor cells with myogenic potential, which aid muscle fiber repair and regeneration in adult tissues. These precursor cells are activated after muscle injury and initiate the healing process by producing new muscle fibers or integrating into the available muscle cells, mainly into their myonuclei, and promoting reparative processes. Satellite cells are devoted to myogenesis and are undifferentiated and mitotically dormant. The only source of new myoblasts in adult tissue is satellite cells, whose numbers decline with age. These cells can become active in ischemic circumstances and behave similarly to bone marrow stem cells in such conditions. Adipose tissue, the umbilical cord, and other sources can also be used to transfer stem cells for therapeutic purposes in addition to bone marrow [30].

Comparison of Intramuscular and Intra-arterial Stem Cell Administration

The route of administration of stem cells for CLI may be IM or IA, or both. Establishing a paracrine cellular depot in the ischemic region is the primary pathophysiology for the intramuscular route. Experimental investigations on animals show that BMDCs can aid in the repair of blood vessels and muscles by physically integrating growth factors into the tissue [31-33], and ischemic tissues can effectively neovascularize when bone marrow mononuclear cells are injected. They induce the regeneration of blood vessels and muscles by paracrine pathways through vascular endothelial cells or direct differentiation of blood vessels and muscles from the precursor cells, which would account for their angiogenic actions [31].

EPCs are among the many cell fractions found in BM-MNCs, and they secrete various angiogenic factors in-vivo. The injected stem cells release a host of angiogenic factors, which improve blood flow and contribute to the incorporation of EPC into the newly formed capillaries, which is most likely how angiogenesis occurs. BMDCs function by physically integrating into the tissue or secreting growth factors to aid in the repair of blood vessels and muscle tissue [34]. The gastrocnemius muscle and blocked native arteries are the best locations for collateral development because of the density of preformed collaterals and the greatest parallel orientation to axially aligned arteries [34].

The effects of either pathway of administration were compared, assessing ABI and the transcutaneous partial pressure of oxygen. Both parameters increased considerably only with intramuscular injection or combination therapy but not with intra-arterial cell therapy alone (TcPO2). However, there was no difference between the distance traveled without discomfort and significantly improved pain level. Unlike experiments using intra-arterial cell therapy, intra-muscle cell therapy significantly improved ulcer healing [35-37].

With the administration of G-CSF, mainly in intramuscular sites, the peripheral blood mononuclear cells were found to be mobilized to the site of ischemic fibers. Clinical signs and symptoms of CLI patients improved considerably though the effects were observed only in a limited number of patients in the pilot investigations [38]. Tateno et al. suggested that the incorporated peripheral blood mononuclear cells stimulate ischemic skeletal muscle cells to form muscle-derived angiogenic factors [39].

Stromal Cell Treatment in CLI

Tateishe-Yuyama et al. [22] explained using autologous stem cells in CLI. Injecting MSCs into the gastrocnemius muscle increases TCO2 and ABI and decreases claudication. 18 of 25 clinical studies have shown good results. Yet, more studies are required to standardize the dosage and route of administration. Using autologous stromal cells becomes challenging in patients with anesthetic difficulties, making bone marrow aspiration impossible. This leads to the necessity of using mesenchymal stem cells as an allogeneic transplant. MSCs are less immunogenic and crossed the phase III trial successfully. The indications of SCT are thromboangitis obliterans [clinical trial showed its efficacy with 4-year follow-up] and atherosclerotic diseases [efficacy of progenitor cell proved in atherosclerotic disease still need a proper protocol for manufacturing, standardization, storage, and delivery of these cells] [22].

Adverse Effects of SCT

Several studies report that after six months of treatment, patients who received BM-MNC experience improved rest pain, pain-free walking period, and tissue oxygen pressure. However, a significant effect was not reported after peripheral blood mononuclear cell injection [22,40]. In addition, studies report a 15% mortality in patients undergoing autologous therapy stem cell implantation for CLI. Recent studies have also demonstrated a high mortality rate in patients with CLI in whom angiogenic cell treatment with IM BM-MNC implantation had been done. However, studies also state that the risk is in the same proportion compared to traditional surgical revascularization procedures [22,40-42].

Hemodialysis, diabetes mellitus, and pre-existing coronary artery disease (CAD) are conditions that severely affect angiogenesis or the ability to salvage limbs, as observed in both animal studies and human environments [43,44].

Role of Auto CD-34 Cell Therapy in CLI

In a double-blinded study, 28 CLI patients underwent cell mobilization with a 5 microgram/kg per day dose of GSF followed by leukapheresis on the fifth day. The CD-34 cells obtained were injected intramuscularly. At 12 months, there was a significant improvement in the mean Rutherford score (reduction in baseline, implying improved patient's limb condition) and a reduced rate of amputation in the cell therapy group compared to control subjects [42].

Role of Neutrophil-lymphocyte Ratio (NLR) in CLI

A simple marker of systemic inflammation acts as a significant prognostic marker for chronic CLI. In a recent study of 561 CLI patients with a median age of 74 were selected, NLR was noted. The study followed the subjects for 31 months, with 162 deaths and 148 major amputations during the study period. The Kaplan-Meier curve shows lower mortality in patients who had NLR <5. There was a significant increase in the rate of deaths and major amputation in patients with NLR >5 [45].

Therapeutic Neovascularization Using Peripheral Blood Mononuclear Cells (PB-MNC) for CLI

The collection of PB-MNC is a very safe and cost-effective mode of stem cell therapy for CLI. 29 patients with CLI from arteriosclerosis obliterans (ASO) or thrombangitis obliterans (TAO) were enrolled in a study where 80% of patients were advised for amputation by their physicians. These subjects underwent PB-MNCs in the ischaemic limbs and were reviewed at 2, 6, and 12 months. The authors noted decreased rest pain and improved claudication. Only three patients underwent non-salvageable procedures [39].

Patients with Limitations of Endovascular and Surgical Revascularization

CLI patients who had undergone previous unsuccessful attempts at revascularization, weak outflow vessels limiting the possibility of surgical revascularization options, or medically unfit patients with substantial co-morbidities that lead to an unacceptable risk of revascularization/endovascular operations are grouped as NO-CLI are Ideal candidates for stem cell/progenitor cell therapy [46,47].

Previously NO-CLI patients had high rates of limb loss and mortality, which were measured using amputation-free survival (AFS). AFS is a composite measure that combines hard outcomes such as amputation and death. It is measured as a one-time event or a cumulative accumulation of events. Stem cell therapy in NO-CLI has resulted in good clinical outcomes, viz., living persons with limbs that have not been amputated

Delivery Methods to Enhance Cell Survival, Paracrine Effects, Engraftment

Various strategies are being employed to enhance the therapeutic effects of stem/progenitor cells. One such strategy is to improve the cell tissue viability of the progenitor cells, mainly achieved through the development of effective cell transport carriers supporting the protective effect of cells or strengthening their overall cell survival. Wang et al. produced a methylcellulose (MC) hydrogel that responds to temperature and enables the temperature-regulated release of placental MSC (P-MSC) at lower temperatures [48]. The thermo-responsive MC-hydrogel as a delivery system for P-MSC in ischemic limbs increased cell survival, improved cell viability, and enhanced blood flow to the limbs, thereby preventing muscle atrophy [49].

Discussion

Surgical or endovascular revascularization has been the first choice of peripheral therapy for obstructive arterial disease (PAOD) and CLI. Up to 30% of patients end up with complications in such procedures due to critical vascular involvement. The prognosis remains equivocal, with mortality rates up to 20% over six months [50,51].

For the last two decades, advances in regenerative medicine have been noted in various fields of modern medicine with proposed stem cell therapies in different forms and varieties. Asahara et al. showed that circulating cells produced in the bone marrow can develop into the endothelium and stimulate endothelial formation, progressing into new blood vessels. These were called EPCs, capable of endothelium and new vessel formation, thereby improving the perfusion of ischemic tissues, especially myocardium and peripheral limb ischemia [52].

SCT is a very promising treatment modality in CLI patients, especially in NO-CLI, where surgical revascularization and endovascular procedures to re-establish the vascular flow have failed or are impossible [53]. In such patients, SCT is a reliable salvage procedure that helps to lower or avoid amputations, increasing ABI and TcPO2 and improving the AFS rate by avoiding or postponing amputations and death [54]. Studies have recorded an overall improvement in ischemic symptoms and their quality of life, especially future diabetic angiopathies and coronary angiopathies.

Aspiration of bone marrow is generally well tolerated. Local pain is the most frequent adverse reaction that can be treated with non-steroidal anti-inflammatory medicines. Even G-CSF stimulation was well tolerated, with myalgia, fever, and bone soreness being the most frequent adverse effects. Intramuscular or intra-arterial administration of BM-MNCs cells has also produced encouraging results; the procedure is safe and well tolerated. If at all any unfavorable reactions were observed, they mostly resulted from some pre-existing disease [55,56].

Given the unique chemokine receptor-4 (CXCR4) crucial for stem cell homing, BM-MSCs appear more effective in initiating reparative processes than mobilized PB-MSCs. Hence, BM-MSC may be a better alternative than PB-MSC. The treatment-induced benefits last for two to three years. A multi-centric, large-scale, randomized control study is required to demonstrate the efficacy and safety of using stem cell injection for PAOD and to establish this therapy as a standard mode of treatment for CLI patients [57].

The ever-increasing trend of molecular approach and linkage between CLI and type 2 diabetes mellitus suggests the need for new medical therapies. miRNAs appear to be favorable therapeutic and diagnostic targets because they are particularly involved in neovascularization [58,59]. All preclinical, animal, and clinical studies point towards an overall positive outcome of such therapy.

Limitations and Challenges of SCT

Despite the very encouraging results of several clinical trials, there are many concerns and barriers to SCT. Furthermore, extensive research is needed to characterize the precise molecular pathways controlling its therapeutic effects. First, we must decide which cell type [BM-MSCs, peripheral blood-derived MSCs, adipose-derived stem cells, induced pluripotent stem cells, umbilical cord-derived MSCs, or dental pulp-derived MSCs] to use. Second, as the stromal cells constitute a heterogeneous population, a deeper comprehension of the effective subset of stem cells is required.

Despite the generally encouraging findings of several clinical trials, there are still many unresolved problems and obstacles to SCT. The specific molecular mechanisms controlling progenitor cell therapeutic benefits are being studied to identify the ideal cell type for cell-based treatment.

Both microvascular and macrovascular issues are related to PAOD/CLI. The current understanding of the mechanism of action of MSCs in CLI is increased microvascular density and stromal cell-mediated angiogenesis to improve the perfusion in CLI limbs. The ideal solution for an ischemic human leg is the production of collateral arteries or, even better, novel blood flow pathways. An in-depth analysis of the mechanisms behind SCT-induced arteriogenesis is required to develop an effective treatment to develop arteriogenesis.

The ideal frequency of application, the best route of administration (IM vs. IA or other targeted delivery), and the right therapeutic dosage of cells are yet to be defined. The impact of autologous versus allogeneic stem cells and various stromal cell varieties must be standardized.

## Conclusions

We conclude that SCT is a promising treatment modality for CLI patients, even in those in whom endovascular and revascularization procedures are not possible. SCT assures a prolonged AFS rate, improved distal perfusion, improved walking distances, reduced amputation rates, and increased survival ratio. Further research is required to characterize the mechanism of action, identify the optimal stem cell variety useful in CLI, uniform routes of administration, and frequency of application, thus standardizing the procedure. Finally, the path from vascular biological principles to effective treatment of CLI requires sustainable optimism, generosity in sharing data and discussing benefits and risks should mark PAD as a treatable condition.
